# DNA Interactions with Ruthenium(ll) Polypyridine Complexes Containing Asymmetric Ligands

**DOI:** 10.1155/BCA.2005.15

**Published:** 2005

**Authors:** Hui Chao, Liang-Nian Ji

**Affiliations:** Department of Chemistry / The Key laboratory of Gene Engineering of Ministry of Eduction Sun Yat-Sen University Guangzhou 510275 China

**Keywords:** Ru(II)complexes; DNA; Asymmetric ligands

## Abstract

In an attempt to probe nucleic acid structures, numerous Ru(II) complexes with different ligands have been synthesized and investigated. In this contribution we focus on the DNA-binding properties of ruthenium(II) complexes containing asymmetric ligands that have attracted little attention in the past decades. The influences of the shape and size of the ligand on the binding modes, affinity, enantioselectivities and photocleavage of the complexes to DNA are described.


### DNA Interactions with Ruthenium(II) Polypyridine Complexes Containing Asymmetric Ligands

Hui Chao, Liang-Nian Ji*

*Department of Chemistry / The Key laboratory of Gene Engineering of Ministry of Eduction, Sun Yat-Sen University, Guangzhou 510275, P. R. China*

**ABSTRACT**

In an attempt to probe nucleic acid structures, numerous Ru(II) complexes with different ligands have been synthesized and investigated. In this contribution we focus on the DNA-binding properties of ruthenium(II) complexes containing asymmetric ligands that have attracted little attention in the past decades. The influences of the shape and size of the ligand on the binding modes, affinity, enantioselectivities and photocleavage of the complexes to DNA are described.

**Keywords:** Ru(II) complexes; DNA; Asymmetric ligands

### 1. INTRODUCTION

Tremendous interest has been evoked by the interactions of substitution-inert metal complexes with nucleic acids over the past decade /1-5/. In particular, ruthenium(II) complexes with polypyridine ligands, due to a combination of easily constructed rigid chiral structures spanning all three spatial dimensions and a rich photophysical repertoire, have attracted considerable attention. Since pioneering studies by Barton and co-workers showed that optically active isomers of $[Ru(phen)_3]^{2+}$ bind to DNA with distinctive characteristics /6/, the binding of ruthenium(II) polypyridyl complexes to DNA has initiated vigorous interest and many structural analogues based on the prototype $[Ru(phen)_3]^{2+}$ have been also synthesized and investigated /1, 7-21/.

However, most of the reported complexes contain symmetric aromatic ligands. Investigations of ruthenium(II) complexes with asymmetric ligands have attracted little attention, and their vast potential as DNA-binding reagents remains largely untapped. In fact, molecular shape, among the various factors that contribute to stabilizing the metal complex on DNA helix, appears to be the most significant. Modification of

* Corresponding author. Tel: +86-20-84036461; fax: +86-20-84035497.
E-mail address: ceschh@zsu.edu.cn the ligand would lead to subtle or substantial changes in the binding modes, location and affinities of the complexes to DNA, making it possible to explore various valuable conformations- or site-specific DNA probes and potential chemotherapeutical agents /6,21,22/. In this review we would like to focus attention on recent progress made in our laboratory regarding the DNA-binding properties of ruthenium(II) complexes containing asymmetric ligands /23-28/.

### 2. MONONUCLEAR RU(II) COMPLEXES CONTAINING ASYMMETRIC LIGANDS

### 2.1. Effect of ligand planarity on the DNA binding affinity

As pointed out previously, modification of the ligand may lead to subtle or substantial changes in the binding modes, location and affinities of the complexes to DNA /21/. The influence of ligand planarity is especially obvious in exploring the DNA binding properties of the series of complexes $[Ru(bpy)_2(ddt)]^{2+}$, $[Ru(bpy)_2(dta)]^{2+}$ and $[Ru(bpy)_2(dpt)]^{2+}$ /28/.

Addition of CT-DNA produced a different extent of perturbation on the absorption spectra of the three complexes; the hypochromism in the MLCT band and other data are listed in Table 1. Intrinsic binding constants $K_b$ of $(2.1 \pm 0.3) \times 10^4$, $(3.7 \pm 0.3) \times 10^4$ and $(6.3 \pm 0.4) \times 10^4$ $M^{-1}$ were obtained for $[Ru(bpy)_2(ddt)]^{2+}$, $[Ru(bpy)_2(dta)]^{2+}$ and $[Ru(bpy)_2(dpt)]^{2+}$, respectively. The viscosity of DNA bound to the three complexes is increased with the increment of the complex concentration (Fig. 1) and has the same trend as observed in spectroscopic titration experiments. The results suggest that $[Ru(bpy)_2(dpt)]^{2+}$ is the most efficient intercalator, $[Ru(bpy)_2(dta)]^{2+}$ next, and $[Ru(bpy)_2(ddt)]^{2+}$ the last. In general, a planar extension of the intercalative ligand would increase the strength of the interaction of the complexes with DNA [2]. As seen in the crystal structure of complex $[Ru(bpy)_2(dpt)]^{2+}$, the two phenyl rings in ddt are rotated away from the 1,2,4-triazine ring with large dihedral angles (45.9° and 42.5°, respectively) (Fig. 2). The significant difference in DNA binding affinity of three complexes can be understood as a result of the fact that the dta and dpt ligands display a more planar conjugate system than that of the ddt ligand.

**Table 1**

The electronic absorption data of Ru(II) complexes upon binding to CT-DNA

| Complex | $\lambda_{max}$/nm | | Binding constant | Ref |
|---|---|---|---|---|
| | $\lambda_{max}$ (nm) | Hypochromism (%) | $K_b/M^{-1}$ | |
| $[Ru(bpy)_2(ddt)]^{2+}$ | 467 | 9.5 | $(2.1 \pm 0.3) \times 10^4$ | [28] |
| $[Ru(bpy)_2(dta)]^{2+}$ | 500 | 13.1 | $(3.76 \pm 0.4) \times 10^4$ | [28] |
| $[Ru(bpy)_2(dpt)]^{2+}$ | 474 | 18.1 | $(6.3 \pm 0.4) \times 10^4$ | [28] |
| $[Ru(tpy)(dppt)]^{2+}$ | 452 | 9.4 | $2.49 \times 10^4$ | [26] |
| $[Ru(tpy)(pta)]^{2+}$ | 485 | 22.5 | $9.51 \times 10^4$ | [26] |
| $[Ru(tpy)(ptp)]^{2+}$ | 506 | 28.1 | $1.62 \times 10^5$ | [26] |
| $[Ru(tpy)(PHBI)]^{2+}$ | 482.5 | 8.0 | $1.6 \times 10^3$ | [27] |
| $[Ru(tpy)(PHNI)]^{2+}$ | 486 | 27.6 | $3.2 \times 10^4$ | [27] |

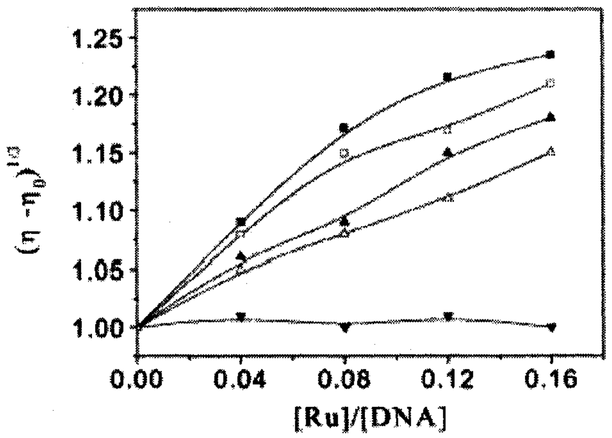

**Fig. 1:** Effect of increasing amounts of the complexes of $[Ru(bpy)_2(dppz)]^{2+}$ (■), $[Ru(bpy)_2(dpt)]^{2+}$ (□), $[Ru(bpy)_2(dta)]^{2+}$ (▲), $[Ru(bpy)_2(ddt)]^{2+}$ (Δ) and $[Ru(bpy)_3]^{2+}$ (▼) on the relative viscosities of calf thymus DNA at 29 (± 0.1) °C (adapted from Ref. [28]).

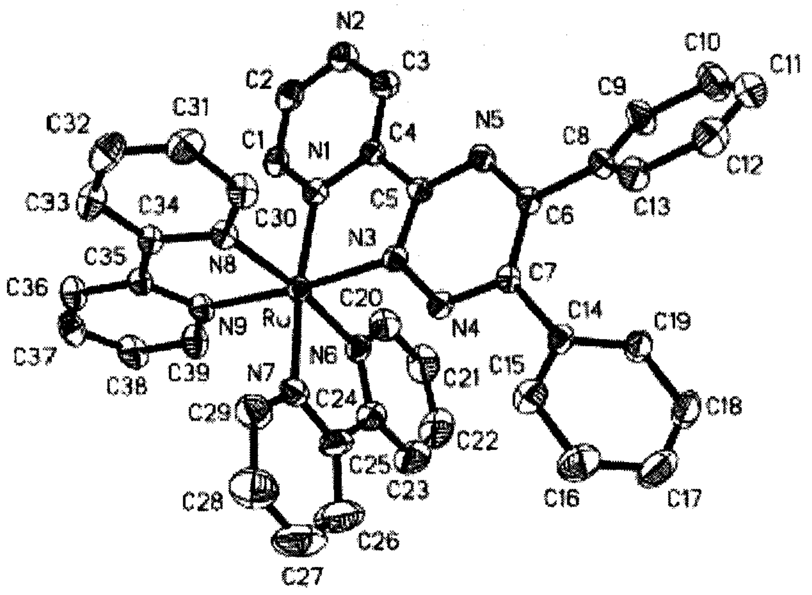

**Fig. 2:** An ORTEP drawing of $[Ru(bpy)_2(dpt)]^{2+}$ (adapted from Ref. [28]).

### 2.2. Effect of ligand shape on the DNA binding geometry

It is expected that increasing the surface area for intercalative stacking by a complex will lead to a substantially increased intercalative binding affinity /2/. However, if the increased part is non-planar relative to the parent ligand, the binding affinity, even the binding mode, may be changed as observed for $[Ru(tpy)(dppt)]^{2+}$, $[Ru(tpy)(pta)]^{2+}$ and $[Ru(tpy)(ptp)]^{2+}$ /26/.

Some electronic absorption data of complexes on binding to DNA are summarized in Table 1. When the amount of DNA is increased, a decrease of 9.4% in the MLCT transitions is found for complex $[Ru(tpy)(dppt)]^{2+}$; for two other complexes, $[Ru(tpy)(pta)]^{2+}$ and $[Ru(tpy)(ptp)]^{2+}$, the decreases are 22.5 and 28.1%, respectively. The intrinsic binding constants $K_b$ were determined as $2.49 \times 10^4$ $M^{-1}$ for $[Ru(tpy)(dppt)]^{2+}$, $9.51 \times 10^4$ $M^{-1}$ for $[Ru(tpy)(pta)]^{2+}$ and $1.62 \times 10^5$ $M^{-1}$ for $[Ru(tpy)(ptp)]^{2+}$ using the MLCT absorption. The data suggest that the interaction of $[Ru(tpy)(ptp)]^{2+}$ with DNA is the strongest, followed by $[Ru(tpy)(pta)]^{2+}$, and then $[Ru(tpy)(dppt)]^{2+}$. The EB competitive binding experiment, which is used to determine the extent of binding between the second molecule and DNA /38,39/, also supports the above trend. The Stern-Volmer quenching constant $K$ values for $[Ru(tpy)(dppt)]^{2+}$, $[Ru(tpy)(pta)]^{2+}$ and $[Ru(tpy)(ptp)]^{2+}$ are 4.89, 27.28 and 30.47, respectively.

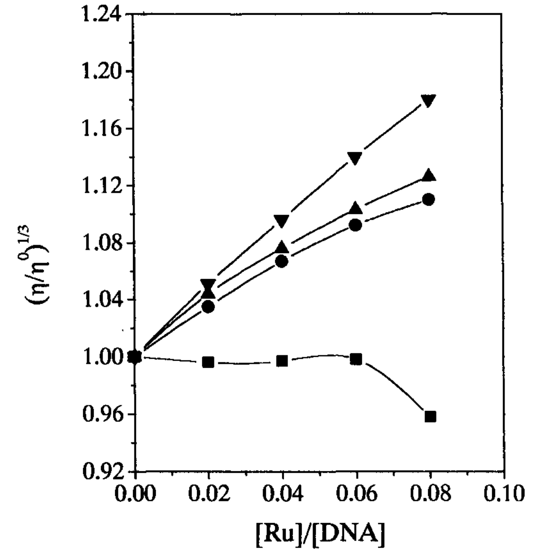

**Fig. 3:** Effect of increasing amounts of the complexes of $[Ru(tpy)(dppt)]^{2+}$ (■), $[Ru(tpy)(pta)]^{2+}$ (●), $[Ru(tpy)(ptp)]^{2+}$ (▲) and $[Ru(bpy)_2(dppz)]^{2+}$ (▼) on the relative viscosities of calf thymus DNA at 30.0 (± 0.1) °C (adapted from Ref. [26]).

This trend can be further testified from viscosity measurements (Fig. 3). $[Ru(tpy)(pta)]^{2+}$ and $[Ru(tpy)(ptp)]^{2+}$ increase the viscosity in a similar fashion to the proven DNA intercalator $[Ru(bpy)_2(dppz)]^{2+}$, but $[Ru(tpy)(dppt)]^{2+}$ exerts essentially no effect on DNA viscosity at low binding ratios; upon further binding of the complex to DNA, the DNA viscosity decreases. This suggests that the three complexes could bind DNA in two different modes: $[Ru(tpy)(dppt)]^{2+}$ in partial, non-classical intercalation mode and $[Ru(tpy)(pta)]^{2+}$ and $[Ru(tpy)(ptp)]^{2+}$ in classical intercalation mode. As revealed by the crystal structures of complexes /25/, in $[Ru(tpy)(dppt)]^{2+}$ the dppt ligand is somewhat sterically hindered from planarity and two phenyl rings are rotated away from the 1,2,4-triazine ring with large dihedral angles (37.2°

and 48.3°, respectively) (Fig. 4), so it does not completely intercalate DNA. The partial intercalation may act as a "wedge" to pry one side of a base-pair stack apart, as observed for the Δ-$[Ru(phen)_3]^{2+}$ /40,41/, but not fully separate the stack as required by the classical intercalation mode. This would cause a static bend or kink in the helix and decrease the viscosity of DNA. On the other hand, for complex $[Ru(tpy)(pta)]^{2+}$ (Fig. 5) and $[Ru(tpy)(ptp)]^{2+}$ (Fig. 6), the two rotated phenyl rings are replaced with a naphthyl ring or a biphenyl ring in pta or ptp ligand, respectively. It is nearly coplanar with 1,2,4-triazine ring and constructs a larger π framework compared to that of dppt. This helps two complexes intercalating into the DNA base pairs deeply and increases DNA viscosity.

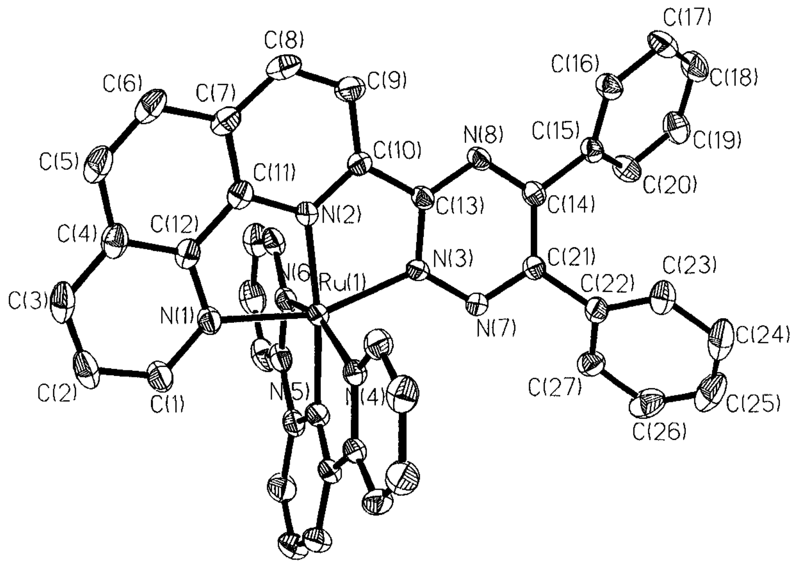

**Fig. 4:** An ORTEP drawing of $[Ru(tpy)(dppt)]^{2+}$ (adapted from Ref. [25]).

Similar cases are also observed in $[Ru(tpy)(PHBI)]^{2+}$ and $[Ru(tpy)(PHNI)]^{2+}$ /27/. The results from optical experiments (Table 1) together with the viscosity measurements (Fig. 7) support that $[Ru(tpy)(PHBI)]^{2+}$ binds to DNA via electrostatic interaction, while $[Ru(tpy)(PHNI)]^{2+}$ binds to DNA by partial intercalation via the extended naphthyl ring into the base pairs of DNA. Although the extended aromatic π-systems in PHBI and PHNI are comparable to those in dppt, pta and ptp, the DNA binding properties of $[Ru(tpy)(PHBI)]^{2+}$ and $[Ru(tpy)(PHNI)]^{2+}$ are different from those of the three complexes discussed above. The great difference in DNA binding mode and binding affinity is attributed to the molecular configuration of complexes. In $[Ru(tpy)(PHBI)]^{2+}$ and $[Ru(tpy)(PHNI)]^{2+}$, tpy and PHBI (or PHNI) sterically compact each other, and complexes with them have relatively shielded surfaces, as seen in $[Ru(bpy)_3]^{2+}$ and $[Ru(phen)_3]^{2+}$. The steric shielding prevents the complexes from intercalating into (or deeply into) the DNA base stack and leads to their low binding affinities.

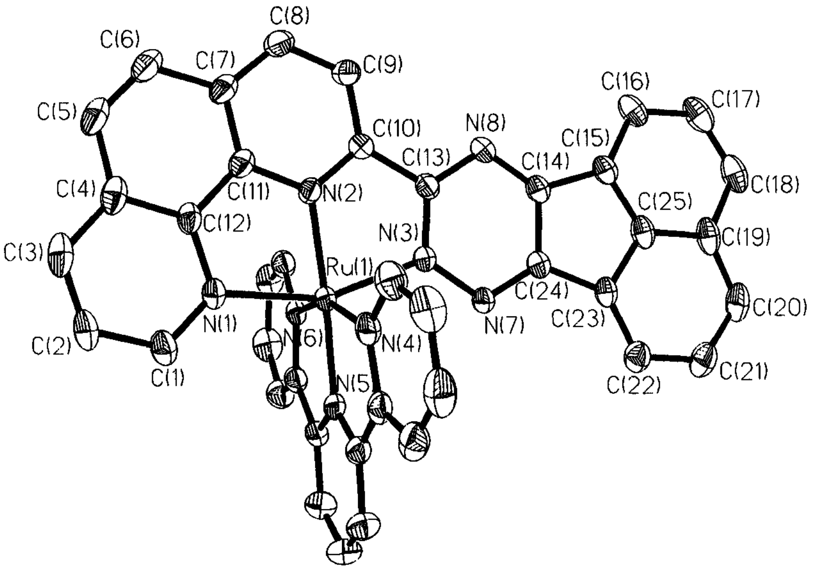

**Fig. 5:** An ORTEP drawing of $[Ru(tpy)(pta)]^{2+}$ (adapted from Ref. [25]).

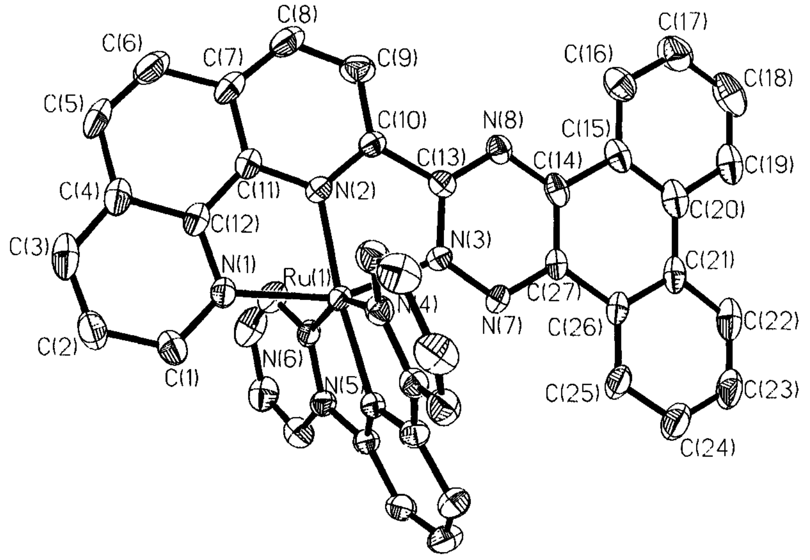

**Fig. 6:** An ORTEP drawing of $[Ru(tpy)(ptp)]^{2+}$ (adapted from Ref. [25]).

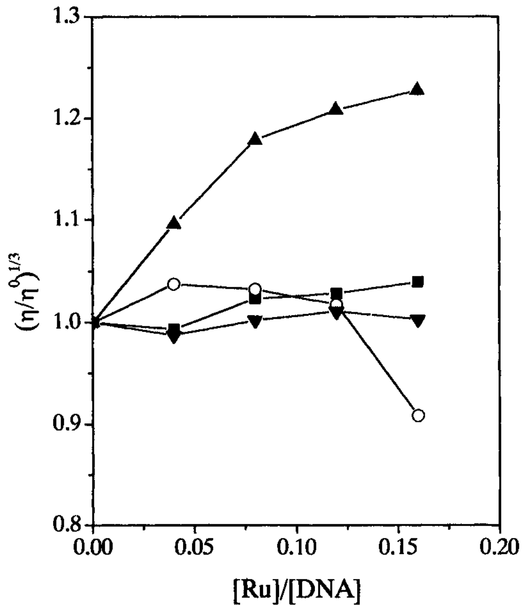

**Fig. 7:** Effect of increasing amounts of $[Ru(tpy)(PHNI)]^{2+}$ (■), $[Ru(tpy)(PHBI)]^{2+}$ (○), $[Ru(bpy)_2(dppz)]^{2+}$ (▲) and $[Ru(bpy)_3]^{2+}$ (▼) on the relative viscosities of CT DNA at 28.0 (± 0.1) °C (adapted from Ref. [27]).

### 2.3. Enantioselective binding to DNA

Equilibrium dialysis experiments may offer the opportunity to examine the enantioselectivity of the complex binding to DNA. According to the proposed binding model [6[, the Δ enantiomer of the complex, a right-handed propeller-like structure, will display a greater affinity than the Λ enantiomer with the right-handed CT-DNA helix, due to the appropriate steric matching.

The CD spectra in the UV region of $[Ru(bpy)_2(dta)]^{2+}$ and $[Ru(bpy)_2(dpt)]^{2+}$ after their racemic solutions were dialysed against CT-DNA are shown in Fig. 8. The dialysate of $[Ru(bpy)_2(dta)]^{2+}$ and $[Ru(bpy)_2(dpt)]^{2+}$ show strong CD signals of Λ enantiomer with a positive peak at 269 and 270 nm, and a negative peak at 287 and 290 nm, respectively. However, the CD spectra for the dialysate of $[Ru(bpy)_2(ddt)]^{2+}$ show no discernible signals. This is related to the difference in the DNA binding affinity of the complexes and also indirectly reflects the influence of ligand shape.

### 2.4. DNA photocleavage studing

The octahedral transition metal complex is stable to the oxidant, but sensitive to light. Upon irradiation, they can cleavage DNA by generating singlet oxygen, or subtracting the hydrogen atom. Some ruthenium(II) complexes have been found to promote the cleavage of DNA [14,15,31-35].

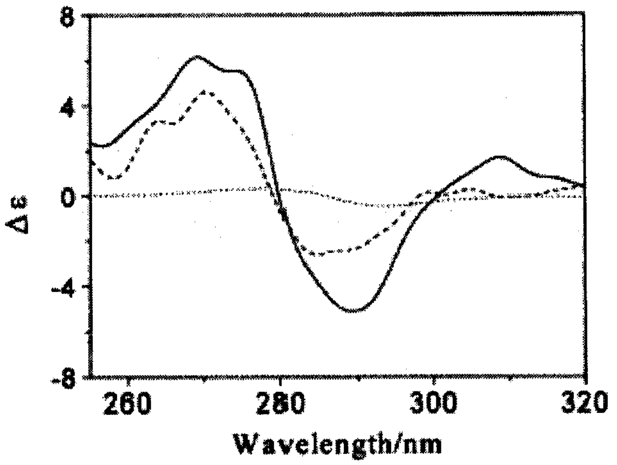

**Fig. 8:** CD spectra of the dialysate of $[Ru(bpy)_2(dpt)]^{2+}$ (solid line), $[Ru(bpy)_2(dta)]^{2+}$ (dash line) and $[Ru(bpy)_2(ddt)]^{2+}$ (dot line) after 48 h of dialysis against CT-DNA ([Ru] = 50 μM, [DNA] = 1.0 mM) (adapted from Ref. [28]).

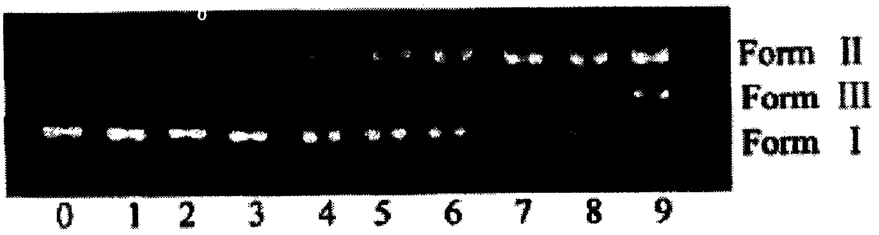

**Fig. 9:** Photograph showing the effect of the ruthenium(II) complexes and light on supercoiled pBR 322 DNA after incubation for 1 h at 37 °C. DNA alone (lane 0), the concentration of $[Ru(bpy)_2ddt]^{2+}$ was 20, 40, 60 μM (lanes 1-3); the concentration of $[Ru(bpy)_2dta]^{2+}$ was 20, 40, 60 μM (lanes 4-6); the concentration of $[Ru(bpy)_2dpt]^{2+}$ was 20, 40, 60 μM (lanes 7-9) (adapted from Ref. [28]).

Fig. 9 shows gel electrophoresis separation of pBR 322 DNA after incubation with three complexes $[Ru(bpy)_2ddt]^{2+}$, $[Ru(bpy)_2dta]^{2+}$ and $[Ru(bpy)_2dpt]^{2+}$ and irradiation at 365 nm. With increasing concentration of the complexes, $[Ru(bpy)_2dta]^{2+}$ and $[Ru(bpy)_2dpt]^{2+}$ caused single-strand nicking with the conversion of form I to form II; the latter complex even induced the double-strand scissions in supercoiled DNA; on the other hand, in the presence of $[Ru(bpy)_2ddt]^{2+}$, no distinct cleavage of pBR 322 DNA is observed. While DNA photocleavage by $[Ru(phen)_3]^{2+}$ has been reported to involve an $^1O_2$-based mechanism /29/, the natures of the reactive intermediates as well as the mechanisms of their actions involved in the DNA photocleavage by these Ru(II) complexes containing asymmetric ligands have not yet been explored in detail. However, the different DNA-nicking efficiencies of these complexes may be related to the absorption intensity at 365 nm and the affinity for DNA.

### 3. DINUCLEAR RU(II) COMPLEXES BRIDGED BY AN ASYMMETRIC LIGAND

Although considerable attention has been mainly devoted to mononuclear ruthenium(II) DNA intercalators over the last decade, there is growing interest in the interaction of dinuclear Ru(II) complexes with DNA /42-51/. For example, Kelly and co-workers have reported that by tethering relatively weak binding systems such as $[Ru(bpy)_3]^{2+}$ into bimetallic systems, binding affinities may be enhanced by several orders of magnitude /41/. Norden and colleagues have reported that non-intercalating bimetallic complexes, in which Ru(II) centers are linked by a semi-rigid dppz dimmer, bind with extremely high affinities /45/. Later they synthesized a bis-intercalating system, where $[Ru(phen)_2dppz]^{2+}$ is tethered together via the dppz moieties and an aliphatic diamide linker /47/. With the aim of developing novel DNA-binding reagents, we investigated the DNA binding properties of $[Ru(bpy)_2(pztp)]^{2+}$ and $[(bpy)_2Ru(pztp)Ru(bpy)_2]^{4+}$ /23/.

The absorption spectrum of $[Ru(bpy)_2(pztp)]^{2+}$ showed a perturbation on addition of CT-DNA, with hypochromism of about 12% in MLCT band. However, upon coordination of another $Ru^{II}$ center in the bridging ligand pztp, the hyperchromism of the MLCT band for $[(bpy)_2Ru(pztp)Ru(bpy)_2]^{4+}$ decreased by 5%, which is only slightly larger than that of $[Ru(bpy)_3]^{2+}$. For further clarification of the interaction between the two complexes with DNA, viscosity measurements were carried out. The effects of $[Ru(bpy)_2(pztp)]^{2+}$, $[(bpy)_2Ru(pztp)Ru(bpy)_2]^{4+}$ and $[Ru(bpy)_3]^{2+}$ on the viscosity of rod-like DNA are shown in Fig. 10. $[Ru(bpy)_2(pztp)]^{2+}$ increases the viscosity of DNA dramatically and nearly linearly at low complex concentration ([Ru]/[DNA] < 0.15). The result suggests that $[Ru(bpy)_2(pztp)]^{2+}$ may bind to DNA by intercalation mode despite its much smaller hypochromism in absorption spectra compared with some known intercalator such as $[Ru(bpy)_2(ppz)]^{2+}$ /52/ and $[Ru(bpy)_2(pip)]^{2+}$ /53/. However, $[(bpy)_2Ru(pztp)Ru(bpy)_2]^{4+}$ decreases the viscosity of DNA dramatically. With two $[Ru(bpy)_3]^{2+}$-like units, the dinuclear complex cannot intercalate between the base pairs of DNA even partially, so it is just an electrostatic binder. Unlike other bimetallic systems, the rigid structure of bridging ligand in $[(bpy)_2Ru(pztp)Ru(bpy)_2]^{4+}$ prevents the further enhancement of DNA bind affinity.

### 4. A NOVEL "MOLECULAR LIGHT SWITCH" FOR DNA

Recently, because of their attractive luminescent properties which are extremely sensitive to the microenvironment, Ru(II) complexes have been used as photoprobes of DNA structures and conformations /1,2/. However, the emission of Ru(II) complexes switched by double strand DNA is rare. $[Ru(bpy)_2(dppz)]^{2+}$ and $[Ru(phen)_2(dppz)]^{2+}$, the most extensively investigated "molecular light switch" for DNA, show no luminescence in aqueous solution but luminesce intensely in the presence of DNA /54/. In our laboratory, two novel complexes $[Ru(pztp)_2(phen)]^{2+}$ and $[Ru(pztp)_2(bpy)]^{2+}$ are found to posses the similar properties /24/. They represent the kind of non-dppz based Ru(II) complex as molecular "light switch" for DNA.

$[Ru(pztp)_2(phen)]^{2+}$ and $[Ru(pztp)_2(bpy)]^{2+}$ do not luminesce in aqueous solution either, but that they emit luminescence at 590 nm and show emission enhancement in the presence of increasing amounts of CT-DNA upon excitation at 462 nm (Fig. 11). On saturation with CT-DNA, the typical relative emission intensities for

$[Ru(pztp)_2(phen)]^{2+}$ and $[Ru(pztp)_2(bpy)]^{2+}$ are 0.07 and 0.35 using the emission of $[Ru(bpy)_3]^{2+}$ in Tris-buffer as a standard.

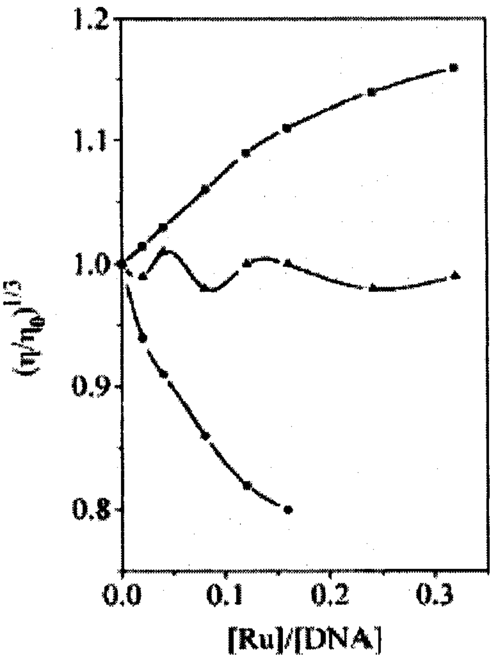

**Fig. 10:** Effect of increasing amounts of $[Ru(bpy)_3]^{2+}$(▲), $[Ru(bpy)(pztp)]^{2+}$(■) and $[(bpy)_2Ru(pztp)Ru(bpy)_2]^{2+}$ (●) on the relative viscosities of CT DNA at 32.7 (± 0.1) °C (adapted from Ref. [23]).

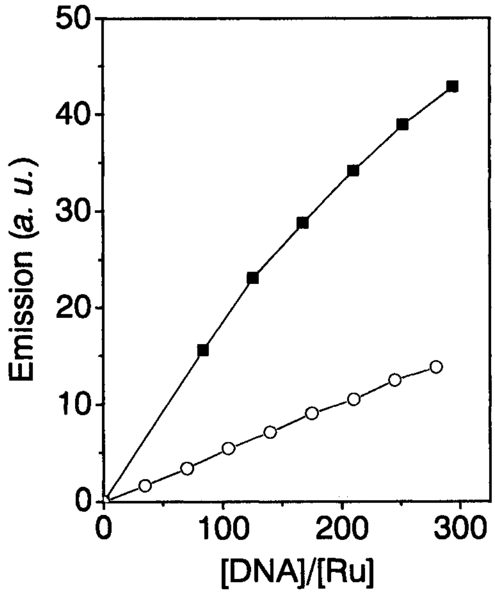

**Fig. 11:** Plots of emission intensity versus [DNA]/[Ru] ratio for $[Ru(pztp)_2(phen)]^{2+}$ (○) and $[Ru(pztp)_2(bpy)]^{2+}$ (■) (adapted from Ref. [24]).

The mechanism of the "light switch" effect for $[Ru(phen)_2(dppz)]^{2+}$ has been studied intensively and accumulated evidence points to hydrogen bonding and/or excited state proton transfer to the phenazine nitrogens as the mechanism of deactivation of the complexes' excited state [54-59].

To explore the possible mechanism involved in the light switch effect, an experiment similar to $[Ru(phen)_2(dppz)]^{2+}$ was carried out. It is noted that the luminescences of $[Ru(pztp)_2(phen)]^{2+}$ and $[Ru(pztp)_2(bpy)]^{2+}$ in $CH_3CN$ are very sensitive to water, being almost completely quenched in the presence of 5% water (v/v). Fig. 12(A) shows the progressive decrease of the emission intensity of $[Ru(pztp)_2(phen)]^{2+}$ in $CH_3CN$ upon the addition of $H_2O$. The titration curves showing the effect of $H_2O$ on the emission of $[Ru(pztp)_2(phen)]^{2+}$ and $[Ru(pztp)_2(bpy)]^{2+}$ in $CH_3CN$ are shown in Fig. 12(B). Similar to that for $[Ru(phen)_2(dppz)]^{2+}$ [31], at low $H_2O$ concentrations, ($[H_2O] < 0.3$ mol dm$^{-3}$), the data fit the Perrin sphere of quenching model very well. Based on Fig. 12(B), the luminescence of $[Ru(pztp)_2(bpy)]^{2+}$ is more sensitive to water than that of $[Ru(pztp)_2(phen)]^{2+}$. These results indicate that the above complexes may experience a similar "light switch" mechanism to that proposed for $[Ru(phen)_2(dppz)]^{2+}$, whose emission is also solvent dependent and displays almost the same trend [59].

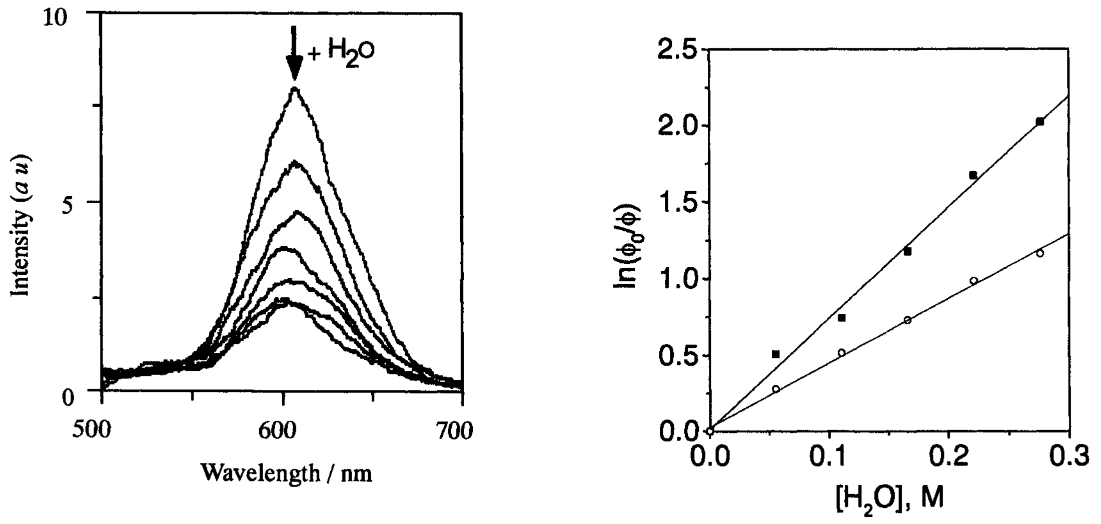

(A) (B)

**Fig. 12:** (A) Emission spectra of $[Ru(pztp)_2(phen)]^{2+}$ in $CH_3CN$ showing the change in intensity with increasing amount of $H_2O$ ([Ru] = 0.2 mmol·dm$^{-3}$, $[H_2O]$ = 0-1.6 mol·dm$^{-3}$). (B) Plots of $\ln(\phi_0/\phi)$ versus $[H_2O]$ for $[Ru(pztp)_2(phen)]^{2+}$ (○) and $[Ru(pztp)_2(bpy)]^{2+}$ (■) (adapted from Ref. [24]).

### ACKNOWLEDGEMENTS

We are grateful to the National Natural Science Foundation of China, the National Science Foundation of Guangdong Province and the Research Fund for the Doctoral Program of Higher Education for their financial support. We also thank the following colleagues for their distinguished contributions to this research field: X.H. Zou, C.W. Jiang, H. Deng, H. Li and W.J. Mei.

### Abbreviations

| | |
|---|---|
| ddt | 3-(pyrazin-2-yl)-5,6-diphenyl-*as*-triazine |
| dta | 3-(pyrazin-2-yl)-*as*-triazino[5,6-*f*]acenaphthylene |
| dpt | 3-(pyrazin-2-yl)-*as*-triazino[5,6-*f*]phenanthrene |
| dppt | 3-(1,10-phenanthrolin-2-yl)-5,6-diphenyl-*as*-triazine |
| pta | 3-(1,10-phenanthrolin-2-yl)-*as*-triazino[5,6-*f*]acenaphthylene |
| ptp | 3-(1,10-phenanthrolin-2-yl)-*as*-triazino[5,6-*f*]phenanthrene |
| PHBI | 2-(2-benzimidazole)-1,10-phenanthroline |
| PHNI | 2-(2-naphthoimidazole)-1,10-phenanthroline |
| pztp | 3-(pyrazin-2-yl)-*as*-triazino[5,6-*f*]1,10-phenanthroline |
| bpy | 2,2'-bipyridine |
| phen | 1,10-phenanthroline |
| tpy | 2,2′:6′,2″-terpyridine |
| phendione | 1,10-phenanthroline-5,6-dione |
| dppz | Dipyrido[3,2-*a*:2',3'-*c*]phenazine |
| EB | ethidium bromide |

### REFERENCES

1. A. Sigel and H. Sigel (Eds.), *Metal Ions in Biological Systems*, vol. 33, Marcel Dekker, New York, 1996.
2. A.M. Pyle and J.K. Barton, *Prog. Inorg. Chem.* **38**, 413 (1990).
3. G. Prativel, J. Bernadou and B. Meunier, *Adv. Inorg. Chem.* **45**, 251 (1998).
4. R.E. Holmlin, P.J. Dandliker, J.K. Barton, Angew. Chem. *Int.* Ed. *Engl.* **36**, 2714 (1997).
5. B. Armitage, *Chem.* Rev. **98**, 1171 (1998).
6. J.K. Barton, *Science* **233**, 727 (1986).
7. K.E. Erkkila, D.T. Odom and J.K. Barton, *Chem. Rev.* **99**, 2777 (1999).
8. C. Moucheron, A.K.D. Mesmaerer and J.M. Kelly, in: M.J. Clarke (Ed.), *Structure and Bonding*, vol. 92, Springer-Verlag, Berlin, 1998, p. 163.
9. Y. Xiong and L.-N. Ji, *Coord. Chem. Rev.* **185-186**, 711 (1999).
10. L.-N. Ji, X.-H. Zou and J.-G. Liu, *Coord. Chem. Rev.* **216-217**, 513 (2001).
11. L.-N. Ji, Q.-L. Zhang and H. Chao, *Chinese* Sci. *Bull.* **46**, 1332 (2001).
12. J.-G. Liu, B.-H. Ye, Q.-L. Zhang, X.-H. Zou, Q.-X. Zhen, X. Tian and L.-N. Ji, J. *Biol. Inorg. Chem.* **5**, 119 (2000).
13. I. Ortmans, C. Moucheron and A. Kirsch-De Mesmaeker, *Coord. Chem.* Rev. **168**, 233 (1998).
14. A. Ambroise and B.G. Maiya, *Inorg. Chem.* **39**, 4256 (2000).
15. A. Ambroise and B.G. Maiya, *Inorg. Chem.* **39**, 4264 (2000).

16. J.G. Collins, A.D. Sleeman, J.R. Aldrich-Wright, I.D. Greguric and T.W. Hambley, *Inorg.* Chem. **37**, 3133 (1998).
17. J.G. Collins, J.R. Aldrich-Wright, I.D. Greguric and P.A. Pellegrini, *Inorg. Chem.* **38**, 3502 (1999).
18. E. Tuite, P. Lincolin and B. Nordén, *J. Am. Chem. Soc.* **119**, 239 (1997).
19. R. E. Holmlin, E.D.A. Stemp and J.K. Barton, *Inorg. Chem.* **37**, 29 (1998).
20. D.Z.M. Coggan, I.S. Haworth, P.J. Bates, A. Robinson and A. Rodger, *Inorg. Chem.* **38**, 4486 (1999).
21. A.M. Pyle, J.P. Rehmann, R. Meshoyrer, C.V. Kumar, N.J. Turro and J.K. Barton, *J. Am. Chem. Soc.* **111**, 3051 (1989).
22. M. Carter, M. Rodriguez and A.J. Bard, *J. Am. Chem. Soc.* **111**, 8901 (1989).
23. X.-H. Zou, B.-H. Ye, J.-G. Liu, Y. Xiong and L.-N. Ji, *J. Chem. Soc., Dalton Trans.* 1423 (1999).
24. X.-H. Zou, B.-H. Ye, H. Li, Q.-L. Zhang, H. Chao, J.-G. Liu and L.-N. Ji, *J. Biol. Inorg. Chem.* **6**, 143 (2000).
25. H. Chao, G. Yang, G.-Q. Xue, H. Li, H. Zhang, I. D. Williams, L.-N. Ji, X.-M. Chen and X.-Y. Li, *J. Chem. Soc., Dalton Trans.*, 1326 (2001).
26. H. Chao, W.-J. Mei, Q.-W. Huang and L.-N. Ji, *J. Inorg. Biochem.* **92**, 165 (2002).
27. C.-W. Jiang, H. Chao, H. Li and L.-N. Ji, *J. Inorg. Biochem.* **93**, 247 (2003).
28. H. Deng, J. Cai, H. Xu, H. Zhang and L.-N. Ji, *J. Chem. Soc., Dalton Trans.* 325 (2003).
29. F.H. Case, *J. Heterocycl. Chem.* **5**, 223 (1968).
30. H. Deng, C.-L. Chen, H. Zhang, C.-Y. Su and L.-N. Ji, *Acta Crystallogr* E. **58**, 01321 (2002).
31. H.-Y. Mei and J.K. Barton, *Proc. Natl. Acad. Sci. U.S.A.* **85**, 1339 (1988).
32. J.M. Kelly, A.B. Tossi, D.J. Mcconnell and C. Ohuigin, *Nucleic Acids Res.* **13**, 6017 (1985).
33. P.K.L. Fu, P.M. Bradley, D. van Loyen, H. Durr, S.H. Bossmann and C. Turro, *Inorg. Chem.* **41**, 3808 (2002).
34. A. Hergueta-Bravo, M.E. Jiménez-Hernández, F. Montero, E. Oliveros and G. Orellana, *J. Phys. Chem. B* **106**, 4010 (2002).
35. W.-J. Mei, J. Liu, K.-C. Zheng, L.-J. Lin, H. Chao, A.-X. Li, F-C. Yun and L.-N. Ji, *J. Chem. Soc., Dalton Trans.* 1352 (2003).
36. K.K. Patel, E.A. Plummer, M. Darwish, A. Rodger and M.J. Hannon, *J. Inorg. Biochem.* **91**, 220 (2002).
37. V.G. Vaidyanathan and B.U. Nair, *J. Inorg. Biochem.* **91**, 405 (2002).
38. B.C. Baguley and M. Lebret, *Biochemistry* **23**, 937 (1984).
39. J. R. Lakowicz and G. Webber, *Biochemistry* **12**, 4161 (1973).
40. S. Satyanarayana, J.C. Dabroniak and J.B. Chaires, *Biochemistry* **31**, 9319 (1992).
41. S. Satyanaryana, J.C. Daborusak and J.B. Chaires, Biochemistry **32**, 2573 (1993).
42. F. O'Reilly, J. Kelly and A. Kirsch-De Mesmaeker, *J. Chem. Soc., Chem. Commun.* 1013 (1996).
43. F.M. O'Reilly and J.M. Kelly, *New J. Chem.* 215 (1998).
44. F.M. O'Reilly and J.M. Kelly, *J. Phys. Chem. B* **104**, 7206 (2000).
45. F.M. Foley, F.R. Keene and J.G. Collins, *J. Chem. Soc., Dalton Trans.* 2968 (2001).
46. P. Lincoln and B. Norden, *J. Chem. Soc., Chem. Comm.* 2145 (1996).

47. B. Önfelt, P. Lincoln and B. Nordén, *J. Am. Chem. Soc.* **121**, 10846 (1999).
48. B. Önfelt, P. Lincoln and B. Nordén, *J. Am. Chem. Soc.* **123**, 3630 (2001).
49. L.M. Wilhelmsson, F. Westerlund, P. Lincoln, B. Nordén, *J. Am. Chem. Soc.* **124**, (12092) 2002.
50. O. van Gijte and A. Kirsch-De Mesmaeker, *J. Chem. Soc., Dalton Trans.* 951 (1999).
51. A. Brodkorb, A. Kirsch-De Mesmaeker, T. J. Rutherford and F. R. Keene, *Eur. J. Inorg. Chem.* 2151 (2001).
52. R.J. Morgan, S. Chatterjee, A.D. Baker and T.C. Strekas, *Inorg. Chem.* **30**, 2687 (1991).
53. J.-Z. Wu, B.-H. Ye, L. Wang, L.-N. Ji, J.-Y. Zhou, R.-H. Li and Z.-Y. Zhou, *J. Chem. Soc., Dalton Trans.* 1395 (1997).
54. A.E. Friedman, J.C. Chambron, J.P. Sauvage, N.J. Turro and J.K. Barton, *J. Am. Chem. Soc.* **112**, 4960 (1990).
55. Y. Jenkins, A.E. Friedman, N.J. Turro and J.K. Barton, *Biochemistry* **31**, 10809 (1992).
56. C.M. Dupureur and J.K. Barton, *J. Am. Chem. Soc.* **116**, 10286 (1994).
57. C. Turro, S.H. Bossmann, Y. Jenkins, J.K. Barton and N.J. Turro, *J. Am. Chem. Soc.* **117**, 9026 (1995).
58. E.J.C. Olson, D. Hu, A. Hörmann, A.M. Jonkman, M.R. Arkin, E.D.A. Stemp, J.K. Barton and P.F. Babara, *J. Am. Chem. Soc.* **119**, 11458 (1997).
59. R.B. Nair, B.M. Cullum and C.J. Murphy, *Inorg. Chem.* **36**, 962 (1997).

